# BRCA1-mediated repression of select X chromosome genes

**DOI:** 10.1186/1479-5876-2-32

**Published:** 2004-09-21

**Authors:** Amir A Jazaeri, Gadisetti VR Chandramouli, Olga Aprelikova, Ulrike A Nuber, Christos Sotiriou, Edison T Liu, H Hilger Ropers, Cindy J Yee, Jeff Boyd, J Carl Barrett

**Affiliations:** 1 From the Center for Cancer Research of the National Cancer Institute. Building 31, Room 3A11, 31 Center Drive, MSC 2440, Bethesda, MD 20892-2440 USA; 2 Max Planck Institute for Molecular Genetics. Ihnestrasse 73, 14195 Berlin Germany; 3 Jules Bordet Institute. Microarray Unit, 121 Bld. de Waterloo, 1000 Brussels, Belgium; 4 Genome Institute of Singapore, 1 Science Park Rd., The Capricorn #05-01, Singapore Sicence Park II 117528, Singapore; 5 From the Departments of Surgery and Medicine of the Memorial Sloan-Kettering Cancer Center 1275 York Ave., New York, New York, 10021 USA

## Abstract

Recently BRCA1 has been implicated in the regulation of gene expression from the X chromosome. In this study the influence of BRCA1 on expression of X chromosome genes was investigated. Complementary DNA microarrays were used to compare the expression levels of X chromosome genes in 18 BRCA1-associated ovarian cancers to those of the 13 "BRCA1-like" and 14 "BRCA2-like" sporadic tumors (as defined by previously reported expression profiling). Significance was determined using parametric statistics with P < 0.005 as a cutoff. Forty of 178 total X-chromosome transcripts were differentially expressed between the BRCA1-associated tumors and sporadic cancers with a BRCA2-like molecular profile. Thirty of these 40 genes showed higher mean expression in the BRCA1-associated samples including all 11 transcripts that mapped to Xp11. In contrast, four of 178 total X chromosome transcripts showed significant differential expression between BRCA1-associated and sporadic tumors with a BRCA1-like molecular profile. All four mapped to Xp11 and showed higher mean expression in BRCA1-associated tumors. Re-expression of BRCA1 in HCC1937 BRCA1-deficient breast cancer cell resulted in the repression of 21 transcripts. Eleven of the 21 (54.5%) transcripts mapped to Xp11. However, there was no significant overlap between these Xp11 genes and those found to be differentially expressed between BRCA1-associated and sporadic ovarian cancer samples. These results demonstrate that BRCA1 mediates the repression of several X chromosome genes, many of which map to the Xp11 locus.

## Introduction

The mechanisms by which mutations in BRCA1 and BRCA2 tumor suppressor genes lead to carcinogenesis are incompletely understood. It remains to be established whether pathways involved in BRCA1 and BRCA2-associated tumorigenesis are also altered in sporadic cancers. Two recent reports demonstrated that BRCA1 and BRCA2-associated tumors have distinct expression profiles in both breast [1] and ovarian [1,2] cancers. With respect to ovarian cancers, two additional novel patterns of gene expression were observed. First, the same set of genes that distinguished BRCA1 and BRCA2-associated tumors also segregated the sporadic (not BRCA1 or BRCA2-associated) ovarian cancers into 2 subgroups consisting of "BRCA1-like" and "BRCA2-like" gene expression profiles. This observation lends support to the hypothesis that the same or similar dichotomous molecular pathways are affected in major subgroups of both hereditary and sporadic ovarian tumors. Second, a disproportionate number of genes located on the Xp11 locus showed statistically significant higher expression in the BRCA1-associated tumors when compared to sporadic tumors. Related to this observation, Ganesan and colleagues recently demonstrated that BRCA1 colocalizes with XIST RNA covering the inactive X chromosome [3]. These investigators showed that repression of BRCA1 led to the increased expression of a green fluorescent protein (GFP) transgene targeted to the inactive X chromosome. However, it remains unknown whether BRCA1 mediates any changes in expression of normal X chromosome genes and whether any such changes are global (affecting the entire X chromosome) or specific to certain genes.

The goal of this study was to investigate further the influence of BRCA1 on the expression of transcripts mapped to the X chromosome. For this purpose the BRCA-associated and sporadic ovarian cancer gene expression data set was analyzed with respect to the expression of 178 transcripts mapped to the X chromosome. Additional in vivo and in vitro experiments employing an X chromosome enriched cDNA microarray were also performed to further evaluate the expression patterns of X chromosome genes in a more comprehensive manner.

## Materials and Methods

### Comparison of gene expression between BRCA-linked and sporadic ovarian cancers

The first part of this investigation consisted of a de novo analysis of the large publicly available data set generated by previous microarray experiments with respect to the 178 X chromosome specific genes [2]. Thus, the description of tumor samples used, BRCA1 and BRCA2 genotyping, tissue processing, and RNA extraction and amplification, and microarray technique were previously published [2]. In addition, detailed protocols describing RNA amplification and microarray hybridization methods are available at  (under "Alternative Methods and Protocols").

### Use of an X-chromosome enriched cDNA microarray for evaluating gene expression differences in BRCA1-associated and sporadic ovarian cancers

For the second part of this investigation a recently developed cDNA microarray enriched in X chromosome transcripts was used. The developmental rationale and approach for this cDNA microarray are described in detail elsewhere [4]. The X-enriched microarray chip used in this investigation consisted of 5,296 features of which 2,879 mapped to the X chromosome. For the purposes of this investigation analysis was limited to only the X chromosome features. Since the cDNAs on this array had not been "sequence-verified" prior to spotting, the cDNA clones for all genes found to be differentially expressed between tumor samples and in cell line experiments were sequenced to ensure positive identification of the transcripts. Those transcripts for which PCR amplification did not result in a product or multiple bands were identified were eliminated from the final analysis.

### Adenovirus-Mediated BRCA1 expression in HCC1937 cells

Tissue culture techniques and adenoviral infection of HCC1937 cells was performed as described previously [5]. Briefly, cells were plated 24 h before the infection at a density 7 x 10^5 ^cells per 100 mm plate. The cells were infected at 250 plaque-forming units per cell with adenovirus encoding full-length human BRCA1 or green fluorescent protein (GFP) cDNAs (the latter used as an irrelevant infection control). Twenty-four hours later cells were harvested and RNA was purified using Trizol Reagent (Life Technologies, Inc.) according to manufacturer's instruction.

### Experiments employing cDNA microarrays

In studies evaluating gene expression differences between ovarian tumor samples using the X-enriched cDNA microarrays, combined RNA from 10 human cell lines (Stratagene, La Jolla, CA) was used as the reference RNA. In studies involving the HCC1937 cell line, gene expression in BRCA1 virally infected cells was directly compared to that of GFP infected controls.

The logarithmic expression ratios for the spots on each array were normalized by subtracting the median logarithmic ratio for the same array. Data were filtered to exclude spots with a size of less than 25 μm, an intensity of less than two times background, or less than 300 units in both red and green channels and to exclude any poor quality or missing spots. In addition, any features found to be missing in greater than 20% of the arrays were not included in the analysis. Statistical comparison between tumor groups was performed with the "BRB Array Tools" software , consisting of a modified *F *test with *P *< 0.005 considered statistically significant. This stringent *P *value was selected in lieu of the Bonferroni correction for multiple comparisons, which was deemed excessively restrictive. For microarray experiments involving the HCC1937 cells infected with BRCA1 or GFP the geometric mean of BRCA1:GFP expression ratio from two separate microarray experiments was used. Genes exhibiting a mean expression ratio change of two-fold or greater were considered significant.

### Quantitative RT-PCR

1 μg of total RNA was reverse transcribed in 50 μl reaction and 5 μl of cDNA was then used for PCR reaction according to Applied Biosystems technical manual. Separate reaction of the same samples with β-actin was performed for normalization purposes. The difference in threshold number of cycles between the ARAF1 and β-actin was then calculated and converted into real fold difference. All measurements were done in triplicates and the results were averaged. Probes for ARAF1 and β-actin were purchased from Applied Biosystems Inc.

## Results

**Figure 1:**
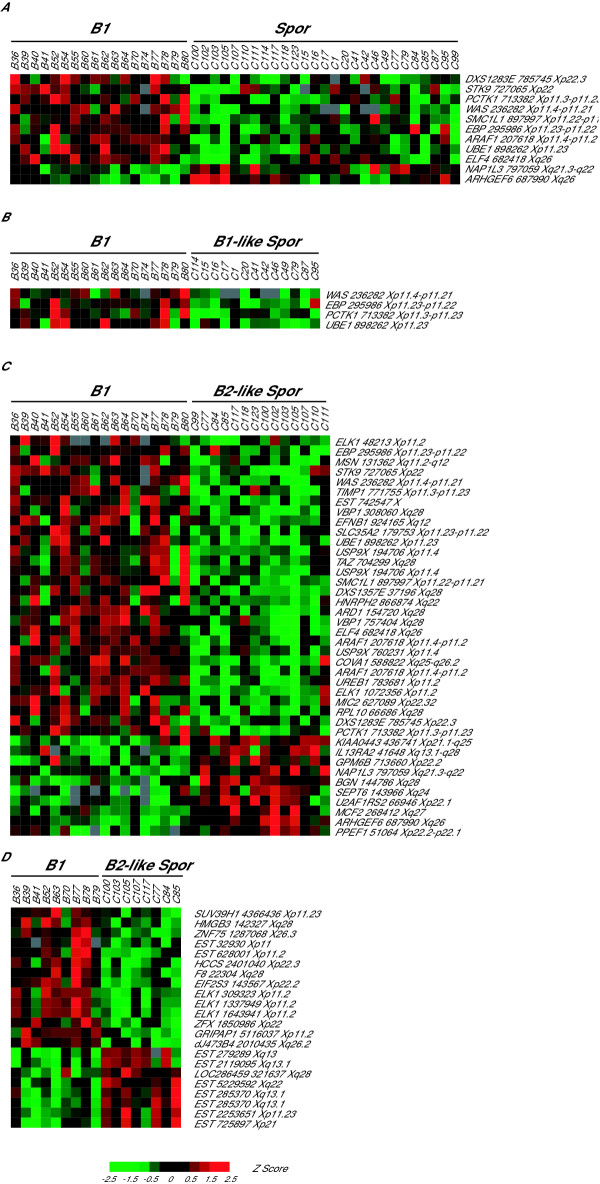
X chromosome gene expression differences BRCA1-associated (B followed by a number) and sporadic (C followed by a number) ovarian cancers (P < 0.005). Genes are shown as hierarchical clusters (using centered correlation and average linkage), samples were not clustered. The red and green color intensities represent expression levels shown as standard normal deviation (Z score) values from each gene's mean expression level (represented as black) across all compared tumor samples. The symbol for each gene is followed by the I.M.A.G.E. clone number of the corresponding cDNA spotted on the array. A. Genes differentially expressed between BRCA1-associated tumors and all sporadic samples. B. Genes differentially expressed between BRCA1-associated cancers and sporadic tumors characterized as "BRCA1-like" based on gene expression profile as described in reference 2. C. Genes differentially expressed between BRCA1-associated tumors and sporadic cancers characterized as "BRCA2-like" based on gene expression profile as described in reference 2. D. An X chromosome enriched cDNA microarray was used to further investigate gene expression differences among a subset of BRCA1-associated and BRCA2-like sporadic tumors. The results of these experiments confirmed the findings observed above in (C).

It was previously shown that when compared to sporadic cancers BRCA1-associated ovarian tumors were characterized by higher mean expression levels of genes mapped to Xp11 [2]. This observation was confirmed when considering all genes mapped to the X chromosome (Fig. 1A). Eleven of the 178 X chromosome mapped transcripts were differentially expressed between the BRCA1-associated and sporadic cancers (P < 0.005). Of these 11 transcripts, six (55%) were located on Xp11. Because the sporadic tumors could be divided into subgroups with "BRCA1- and BRCA2-like" expression profiles [2], one may anticipate differences in the expression levels of genes mapped to Xp11 when comparing each of these sporadic cancer subgroups to the BRCA1-associated tumors. The expression levels of X chromosome genes in the 18 BRCA1-associated cancers was compared to those of the 13 BRCA1-like and 14 BRCA2-like sporadic tumors (Fig. 1A,1B,1C,1D). Only 4 genes showed significant differential expression between the BRCA1-associated and the sporadic tumors with a BRCA1-like molecular profile (Fig. 1B). All 4 mapped to Xp11 and showed higher mean expression in BRCA1-associated tumors. In contrast, 40 of the 178 X-chromosome transcripts were differentially expressed between the BRCA1-associated tumors and sporadic cancers with a BRCA2-like molecular profile (Fig. 1C top panel). Thirty of the 40 transcripts showed higher mean expression in the BRCA1-associated samples including all 11 genes that mapped to Xp11 (Fig. 1C top panel). These data suggest that BRCA1 may be involved in the regulation of gene expression from the X-chromosome. Furthermore, there appears to be a role for BRCA1 in suppressing the expression of several genes mapped to the Xp11 locus that were all higher expressed in BRCA1-associated tumors. This pattern of expression was observed when the BRCA1-associated samples were compared to all sporadic cancers regardless of their expression profile characterization as BRCA1- or BRCA2-like.

The significance of this differential pattern of gene expression between the BRCA1-associated and sporadic cancers is unclear at this time. However, higher expression from Xp11 may be related to the earlier age of presentation of epithelial ovarian cancers in BRCA1 mutation carriers compared to tumors in BRCA2 mutation carriers and patients with sporadic ovarian cancer [6]. This observation cannot be solely explained by an earlier occurrence of a "second hit" as modeled by Knudson's two-hit hypothesis [7] because the age of presentation of ovarian cancer in BRCA2 tumors is no different than that observed in patients with sporadic epithelial ovarian cancers [2,6], thus indicating a BRCA1-specific effect on the age of presentation.

**Figure 2:**
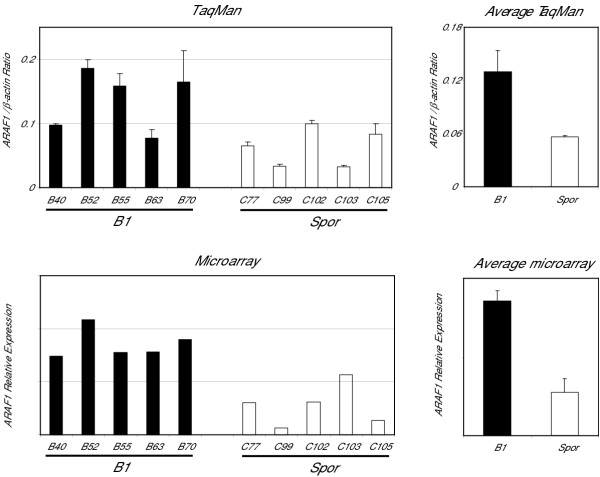
Quantitative RT-PCR evaluation of ARAF1 expression confirms cDNA microarray data. Average of three RT-PCR repeats for each sample is shown on top left panel. ARAF1 expression levels derived by cDNA microarray relative to universal reference RNA is shown in the bottom left panel. Mean expression levels are graphed to the right; error bars represent standard error of mean.

In order to confirm and expand on the above noted differences in gene expression between BRCA1-associated and sporadic tumors, an X-chromosome enriched cDNA microarray was used [4]. Due to the limited availability of resources this cDNA microarray was used to evaluate gene expression in a representative subset of 9 BRCA1-associated and 8 sporadic tumors. The sporadic tumors were selected from the subgroup with a "BRCA2-like profile" as these samples showed more robust differences in X chromosome gene expression in the above noted experiments using our conventional cDNA microarray (Fig. 1B and 1C). Twenty-one transcripts showed significant differential expression between the BRCA1-associated and sporadic tumors with a BRCA2-like profile (P < 0.005). Consistent with our earlier findings and despite the smaller number of samples used for this comparison, the majority of transcripts exhibited higher mean expression in BRCA1-associated samples including all but one of the transcripts located on Xp11 (Fig. 1D). This pattern of expression was confirmed using quantitative RT-PCR of the ARAF1 gene in a representative sample of BRCA1-associated and sporadic ovarian cancer samples (Fig. 2).

**Figure 3:**
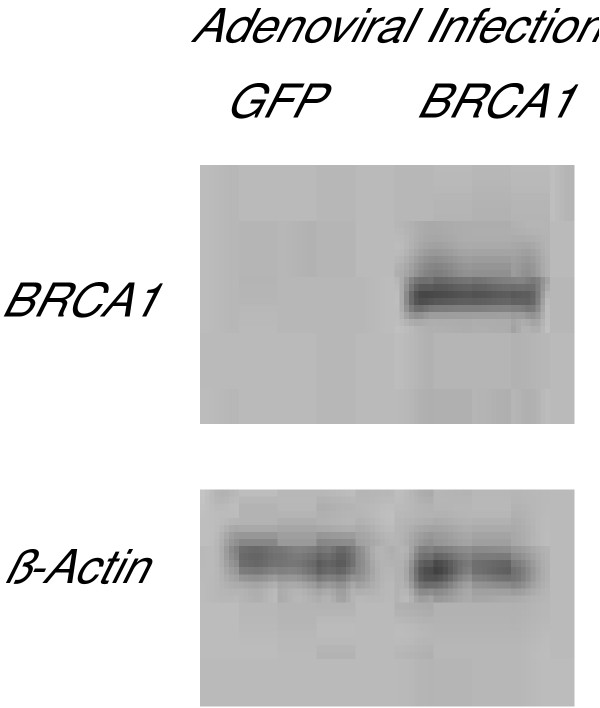
Adenovirus-mediated BRCA1 expression in HCC1937 cells.

We next sought to determine if differences in X chromosome gene expression between BRCA1-associated and sporadic tumors were directly mediated by BRCA1 as oppose to other, possibly confounding, features of these tumors. The HCC1937 breast cancer cells that are either homozygous or hemizygous for the BRCA15382insC mutation were used as a model. Gene expression in HCC1937 cells following virally mediated expression of wild-type BRCA1 was compared to gene expression following viral infection of GFP which was employed as an irrelevant infection control. Prior to using the X-chromosome enriched array, the validity of this approach was tested using a 7.5 K microarray whose features included BRCA1. This preliminary experiment demonstrated that viral infection did in fact result in a 3.4 fold higher BRCA1 expression compared to the GFP control (data not shown). BRCA1 expression was also demonstrated using Western blotting which confirmed BRCA1 protein expression following viral infection (Fig. 3).

Twenty-one X-chromosome transcripts demonstrated at least a two-fold mean expression change across two independent experiments following BRCA1 infection. It is notable that all 21 transcripts (representing 16 unigene clusters) showed decreased expression following BRCA1 transfection with a median repression of 2.4 fold. This uniform repression is particularly significant because median based normalization of logarithmic ratios was employed in microarray data analysis. Thus, the median log expression ratio for all 5,296 features on the array was adjusted to zero (corresponding to an expression ratio of 1.0). Following this median based normalization, the median log expression ratio of the transcripts mapped to the X chromosome (2,879 of the total 5,296 features on the array) was also very close to zero (median log_2 _ratio = 0.016 corresponding to an expression ratio of 1.01). Thus, the observed down regulation of genes does not appear to be a result of the X-enriched composition of the cDNA microarray used in these experiments nor can it be explained in terms of a global down regulation of all X chromosome transcripts. Rather, the observed repression appears to be a specific effect of BRCA1 expression on these 21 transcripts.

Eleven of the 21 (52.4%) transcripts map to Xp11 and represent 8 unigene clusters (Table 1). The most highly repressed (11.6-fold) transcript was that of LOC139135 (Hs.160594) whose protein product contains a PAS domain and is weakly similar to the circadian locomoter output cycles kaput (CLOCK) protein of human and several other organisms. The PAS domain is a highly conserved motif essential for sensing changes in a variety of different environmental conditions including light, oxygen tension and redox potential [8,9]. HIF-1 alpha and EPAS-1, two other PAS containing proteins, are upregulated in a number of human tumors and play an important role in angiogenesis by stimulating VEGF expression [9-11]. LOC139135 deserves additional investigation of its role as a possible protooncogene subject to regulation by BRCA1.

**Table 1:** X chromosome genes showing two-fold or greater change following BRCA1 expression in HCC1937 BRCA1-null cells.

Name	Clone ID*	Locus	Unigene	Fold repression	Comments
LOC139135	C03503	Xq28	Hs.160594	11.6	Similar to CLOCK protein
EST	32930	Xp11	Hs.99070	8.9	
EST	34280	Xp11.2	Hs.99070	8.5	
CSTF2	1705354	Xq22.1	Hs.693	5.5	
EST	1535341	Xq13	Hs.444962	5.4	
EST	1614299	Xq13	Hs.197801	4.3	
JM11	1913391	Xp11.23	Hs.417068	3.5	
ZNF6	1564783	Xq13	Hs.326801	3.0	
ZNF6	5201496	Xq13	Hs.326801	2.4	
LOC158572	2070337	Xp11.23	Hs.408191	2.8	
LOC158572	1880263	Xp11.2	Hs.408191	2.4	
LOC158572	470925	Xp11.23	Hs.408191	2.1	
EST	4402168	Xq26	Hs.175894	2.6	
KLF8	2148451	Xp11.21	Hs.411296	2.5	
KLF8	2148451	Xp11.21	Hs.411296	2.3	
EST	2516780	Xp11.23	Hs.293317	2.4	Moderately similar to PAGE-5 protein
EST	2659258	Xq28	Hs.312560	2.2	
ED1	2030638	Xq12-q13	Hs.105407	2.2	Weakly similar to PAGE-5 protein
TIMP1	172210	Xp11.23	Hs.446641	2.1	
EST	2111889	Xp11.23	Hs.163473	2.0	Weakly similar to XAGE-5 protein
FLJ23614	1938584	Xq26	Hs.28780	1.9	

*Integrated **M**olecular **A**nalysis of **G**enomes and their **E**xpression (I.M.A.G.E.) Consortium cDNA clone identification.

Two transcripts representing the same unigene cluster, Hs. 99070, showed greater than 8 fold repression following BRCA1 expression in HCC1937 cells (Table 1). In addition, an EST (clone ID 32930) belonging to this same unigene cluster was found to be significantly higher expressed in BRCA1-associated ovarian cancers compared to sporadic tumors (Fig. 1D). As such this gene may be one potentially important target of BRCA1 regulation of gene expression from the X chromosome. There was no other overlap between the list of genes differentially expressed following BRCA1 expression in HCC1937 cells and the list of genes differentially expressed between BRCA1-associated and sporadic ovarian cancers. The lack of a broader overlap between the list of genes repressed following BRCA1 expression in the HCC1937 and those differentially expressed between BRCA1-associated and sporadic ovarian cancers is notable. This signifies that BRCA1's influence over transcription is unlikely to be gene specific and rather may involve more global influences over transcription such chromatin remodeling and changes in methylation states.

BRCA1 expression led to the down regulation of several ESTs homologous to PAGE-5, a member of the cancer-testis antigen group of genes (MAGE, GAGE, PAGE, etc.). These ESTs are likely to represent as yet undiscovered members of this family of genes that are known for their characteristic pattern of expression, usually limited to the testes and tumors [12,13]. Intriguing parallels exist between expression characteristics of cancer testis antigens and expression changes mediated by BRCA1. The vast majority of cancer-testis antigen genes are located within discrete loci on the X chromosome [13]. Our results demonstrate that BRCA1 represses the expression of clusters of genes on Xp11, Xp21-p22, Xq13, and Xq26-q28 (Table 1), which correspond to the genomic location of several major cancer testis antigen gene clusters [12,13]. Furthermore, high expression of BRCA1 in pachytene spermatids [14,15] correlates with a significant down regulation of at least one cancer testis antigen, MAGE-B4 [16]. Finally, recent reports have documented the aberrant expression of several cancer-testis antigens in a significant portion of ovarian cancers and linked their expression to drug-resistance [17,18].

Although not completely understood, the expression of cancer testis antigens is thought to be, at least partially regulated by DNA methylation [12]. These data point to changes in DNA methylation as another possible mechanism involved in the BRCA1-mediated repression of gene clusters within the X chromosome. Further investigation of the sequence and genomic organization of genes in these loci will be useful for elucidating features responsible for the co-regulation of these genes.

## Discussion

Whether any of these BRCA1-regulated X chromosome genes are involved in ovarian carcinogenesis and / or tumor progression remains to be determined. However, several lines of evidence support a possible connection between the X chromosome and ovarian neoplasia. First, the loss of a number of regions in the X-chromosome has been associated with ovarian agenesis or premature ovarian failure, which are commonly observed in Turner syndrome and related disorders [19,20]. Thus, the X chromosome is likely to contain genes involved in ovarian maintenance [19]. Aberrant overexpression of such potential ovarian survival/growth regulators on the X chromosome through a mechanism involving the loss of BRCA1 may be involved in ovarian carcinogenesis and/or tumor progression. Chromosome X alterations have been reported in sporadic ovarian carcinomas and borderline tumors [21,22]. Non-random X inactivation has been reported in populations of hereditary ovarian and breast cancer syndrome patients including BRCA1 mutation carriers [23,24]. Finally, in a comparison of gene expression between matched primary and recurrent chemoresistant ovarian cancer samples from the same patient XIST was the most differentially expressed gene and its expression was negatively correlated with response to paclitaxel chemotherapy [25].

Until recently, a mechanistic explanation for how BRCA1 may affect the expression of multiple genes on the X chromosome was lacking. Evidence for the existence of one such mechanism has been provided by Ganesan and colleagues who have demonstrated the co-localization and interaction between XIST and BRCA1 [3]. This interaction was shown to be sufficient and necessary to repress the expression of a green fluorescent protein transgene introduced into the inactive X chromosome. Our investigation shows that genes endogenous to the X chromosome are also repressed by BRCA1 and that genes on certain loci are preferentially affected. The exact nature of this interaction and possible differential effects on gene expression from various regions of the X chromosome remain to be determined. It is unclear whether BRCA1's effect involves changes in XIST RNA expression. Ganesan et al. did not observe such an effect, but other investigators have reported a two-fold increase in XIST RNA levels following BRCA1 expression [26]. Using an X chromosome enriched microarray that has previously been shown to be able to detect changes in XIST expression associated in X chromosome polysomies [4], no increase in XIST RNA was observed following BRCA1 expression. BRCA1 may target XIST in such a way as to bring about changes in the expression of various loci on the X chromosome. It is also possible that BRCA1 may be acting independent of XIST through a different mechanism such regulation of DNA methylation. Alterations in DNA methylation play an integral role in the normal process of X chromosome inactivation and are also involved in the characteristic expression of cancer-testis antigens most of which reside on the X chromosome as discussed above.

One of the unexplained features of germ-line BRCA1 mutations is the overwhelmingly disproportionate risk of cancer in female carriers. One hypothesis put forth to explain this observation is that estrogen is the inciting event by leading to deregulated proliferation and carcinogenesis in hormonally responsive tissues [27]. An alternative, non-mutually exclusive, hypothesis is that the deregulated expression of an X-linked gene normally under BRCA1 control may play a role in predisposing women to carcinogenesis. This a plausible scenario if BRCA1 proves to be involved in the process of X-chromosome inactivation and /or gene dosage regulation for those genes on the X chromosome that do not undergo inactivation. Accordingly, the lack of a need for X chromosome inactivation and X-linked gene dose adjustment in men may explain why male BRCA1 mutation carriers do not have the same increased risk for cancers. Future studies will be aimed at testing these hypotheses.
